# Important Considerations for Sample Collection in Metabolomics Studies with a Special Focus on Applications to Liver Functions

**DOI:** 10.3390/metabo10030104

**Published:** 2020-03-12

**Authors:** Lorraine Smith, Joran Villaret-Cazadamont, Sandrine P. Claus, Cécile Canlet, Hervé Guillou, Nicolas J. Cabaton, Sandrine Ellero-Simatos

**Affiliations:** 1 Toxalim (Research Center in Food Toxicology), Université de Toulouse, INRAE, ENVT, INP-Purpan, UPS, 31300 Toulouse, France; lorraine.smith@inrae.fr (L.S.); joran.villaret-cazadamont@inrae.fr (J.V.-C.); cecile.canlet@inrae.fr (C.C.); herve.guillou@inrae.fr (H.G.); nicolas.cabaton@inrae.fr (N.J.C.); 2 LNC Therapeutics, 17 place de la Bourse, 33076 Bordeaux, France; sandrine.claus@lnc.bio

**Keywords:** metabolomics, standard operating procedures, urine, blood, feces, tissue, cells, liver function

## Abstract

Metabolomics has found numerous applications in the study of liver metabolism in health and disease. Metabolomics studies can be conducted in a variety of biological matrices ranging from easily accessible biofluids such as urine, blood or feces, to organs, tissues or even cells. Sample collection and storage are critical steps for which standard operating procedures must be followed. Inappropriate sample collection or storage can indeed result in high variability, interferences with instrumentation or degradation of metabolites. In this review, we will first highlight important general factors that should be considered when planning sample collection in the study design of metabolomic studies, such as nutritional status and circadian rhythm. Then, we will discuss in more detail the specific procedures that have been described for optimal pre-analytical handling of the most commonly used matrices (urine, blood, feces, tissues and cells).

## 1. Introduction

Metabolomics refers to the high-throughput quantification and characterization of small molecules (metabolites) in tissues or biofluids. Such biochemical profiles contain latent information relating to inherent parameters, such as the genotype, and environmental factors, including the diet, exposure to xenobiotics and gut microbiota. The liver is the heaviest organ in the human body, with a wide array of functions that can be divided into intermediary metabolism (including a central role in carbohydrate, lipid and nitrogen metabolism), immunological activity, secretion of bile, synthesis of various serum proteins, degradation of hormones, and detoxification of xenobiotics. Hepatic lipid catabolism plays a crucial role during fasting and/or prolonged exercise. Upon lowering of blood glucose, the liver increases glucose production by augmenting gluconeogenesis and glycogenolysis to maintain blood glucose levels; increases fatty acid oxidation and ketogenesis to provide extra-hepatic tissues with ketone bodies; and decreases lipogenesis to attenuate triglyceride storage. In the fed state, the opposite occurs and the liver increases glucose uptake to feed glycogenesis; limits lipid oxidation to favor lipogenesis and promotes saving of fatty acids in the form of triglycerides that are packaged in lipoproteins for remote storage in the white adipose tissue. Hence, the liver plays an essential role in the regulation of energy metabolism. Dysregulation of these metabolic pathways leads to metabolic diseases among which non-alcoholic fatty liver disease (NAFLD), which is diagnosed when more than 5% of hepatocytes are steatotic in patients who do not consume excessive alcohol. The disease severity ranges from simple steatosis to steatohepatitis, advanced fibrosis and cirrhosis. NAFLD epidemic represents a major public health burden [1] and remains an unmet medical need [2].

Metabolomics has found numerous applications in the study of liver functions in health and disease. Among others, these include: Non-invasive biomarker investigations to discriminate between the different stages of progression of NAFLD using non-invasive biofluids (urine and plasma) [3,4]; investigation of mechanisms underlying hepatic disease progression such as acute-on-chronic liver failure using serum metabolic profiling [5] or fibrosis [6]; characterization of the gut microbiota metabotypes in urine of NAFLD patients [4,7]; nutrimetabolomics studies to unravel hepatic pathways dysregulated directly in liver samples upon various nutritional challenges [8,9]; discovery of new metabolic functions for nuclear receptors, that are important regulators of liver physiology using direct hepatic metabolomics or other informative fluids such as urine and bile [10,11,12,13]; identification of patients at risk for idiosyncratic drug-induced liver injury (IDILI) before drug administration, a concept named “pharmaco-metabolomics”, that was first demonstrated in urine of animal models [14] and is now extended to human biofluids (urine and serum) [15,16]; study of mechanisms of action for pharmaceutical drugs in urine and fecal samples [17] and environmental contaminants in HepG2 cells and animal biofluids and tissues [18,19].

There are many sources of variation in metabolomic studies, some of which are directly related to the pre-analytical handling steps. Pre-analytical questions are indeed a crucial part of metabolomics study designs since inadequate sample collection, pre-treatment or storage can significantly affect sample quality and result interpretation. The reliability of the metabolomics approach requires inactivation of ongoing metabolism, metabolite stabilization and maintenance of sample integrity. In consideration of this, it is useful to have standard operating procedures (SOP) for pre-analytical handling of samples before starting a metabolomic study. In this review, we discuss the influence of sample collection pre-analytical handling procedures and storage conditions on the metabolomic profiles of the biological matrices that are most commonly used to investigate liver functions, namely urine, blood, feces, tissues and cells. Of note, metabolic profiling of several other matrices such as bile and ascitic fluids can provide interesting information about liver functions [10,20,21] but will not be further discussed in this review given the paucity of data regarding sample collection and stability.

## 2. Overview of the Pre-Analytical Handling Procedures of the Most Commonly Used Biological Matrices in Metabolomics

### 2.1. Time of Collection

#### 2.1.1. Considering Nutritional Status

The choice of time of collection is a crucial step for a successful metabolomics study and will depend on the research question under examination. Nutritional status of the experimental subjects greatly influences the circulating, urinary, fecal and tissue metabolomes and has to be carefully chosen. If one aims to identify a biomarker specifically associated with a food item, then acute postprandial urine will certainly be collected. Criteria for good biomarkers of habitual nutritional intake are metabolites that are metabolically inert and rapidly absorbed within 1.0–1.5 h of consumption in the upper gastrointestinal tract. Such markers are subsequently excreted 1.5–2.5 h later [22]. Plasma is more reflective of modulations in endogenous metabolism as a result of the food metabolome and it should be noted that perturbations of the plasma metabolic profile arise when homeostatic function is impaired. Therefore, fasting plasma samples are usually used to explore how systemic metabolism differs between populations with different dietary habits [23]. Of note, in rodents, 16-h fasting has been shown to affect 1/3 to 1/2 of monitored serum metabolites, with an increase in fatty and bile acids and a significant decrease in diet- and gut microbiota-derived metabolites [24]. Nutritional status also has a significant effect on the tissue metabolome. Especially in the liver, 77% of the hepatic metabolome has been shown to be sensitive to a nutritional high-fat-diet challenge at all times of day. Amino acids, xenobiotics and nucleotides were especially affected and decreased in HFD-fed mice at all time points [25]. Finally, the fecal (or cecal) metabolome is increasingly considered as a functional readout of the gut microbiome and can be used as an intermediate phenotype mediating host–microbiome interactions [4,26]. Although the microbial metabolome still represents an analytical challenge and many microbial metabolites still remains unknown, it is known that the fecal metabolome is highly sensitive to nutritional challenges [27,28] and influences the host hepatic metabolism [4,29,30].

#### 2.1.2. Considering Circadian Rhythm

Circadian rhythms govern a large variety of behavioral, physiological and metabolic processes [31]. Recent advances reveal that a very large fraction of mammalian metabolism undergoes circadian oscillations. Many metabolic pathways are under circadian control and, in turn, may feedback to the clock system to assist in circadian timekeeping [32]. Transcriptomics studies have extensively illustrated a substantial fraction of the genome controlled by the molecular clock [33]. Metabolomics studies have also highlighted the circadian oscillations of metabolites in humans independently of sleep or feeding [34]. In mice, more than 40% of the serum metabolome and 45% of the liver metabolome have been shown to be sensitive to time, with both matrices providing different and complementary information. For example, more than 30% of the serum lipids were not found in the liver and more than half of them oscillated across the circadian cycle, while only 30% of the hepatic lipids oscillated [25]. Moreover, a high-fat challenge induced a loss of serum metabolite rythmicity, compared with the liver [25]. Therefore, when collecting samples for a metabolomics study, one should be aware that a tissue-specific and time-dependent disruption of metabolic homeostasis exists independently of feeding, but also in response to altered nutrition. Time of collection therefore needs to be carefully chosen, and if sample collection is spread between several days, time of collection should be homogenous between the collection days [35].

### 2.2. Common Sources of Variation in Pre-Analytical Handling of Main Biological Matrices

Specific SOPs have been described for collection, preparation and storage of metabolomics samples and will be described along the specificity of each biological matrix in the following sections. Several features of the pre-analytical steps are however shared between the different matrices. First, the numbers, weights or volumes of the samples are important points to anticipate before collection. Second, during collection, samples have to be kept at the lowest temperature possible, and immediate snap freezing is recommended in order to quench any rapid degradation activity such as oxidation of labile metabolites as well as enzymatic reactions.

**Figure 1:**
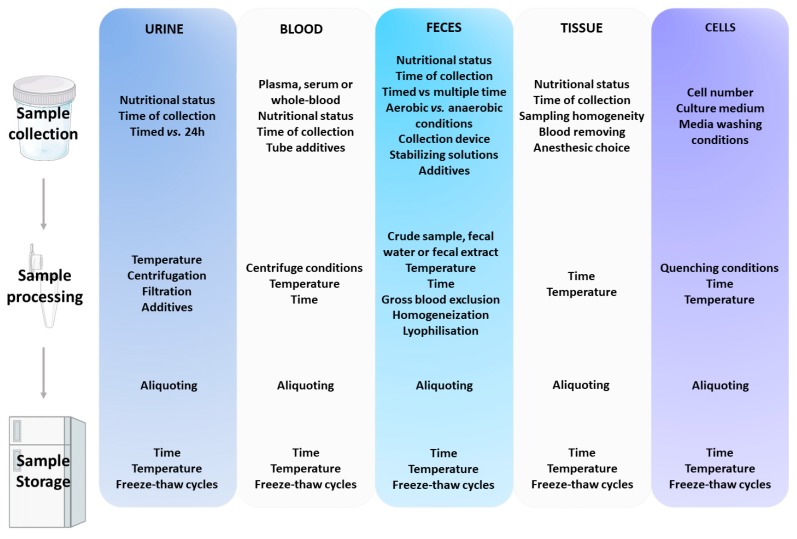
Summary of pre-analytical factors that can affect metabolite profiles in various matrices.

Third, aliquoting the samples should also be considered whenever possible. This important step will avoid repeated freeze–thaw cycles that lead to progressive loss in sample quality. Finally, long term storage at −80 °C or less is recommended before analysis. These general recommendations, as well as matrix-specific pre-analytical factors that influence the results of metabolomics studies are summarized in Figure 1.

### 2.3. Urine

Urine is a biofluid commonly used for both human and animal metabolomics studies because sample collection is non-invasive. The simplicity of the collection allows multiple collections for kinetic studies and ensures reliability of the analysis. Urinary profiles contain signals derived from both endogenous and environmental sources, including diet and gut microbiota metabolic activity, and can therefore provide an overall measure of the metabolic phenotype. It is a collection of waste and biological by-products that reflects a large panel of metabolic processes that may have occurred over time and provides the researcher with a historical overview of the global metabolic events. In addition, it may contain cells (erythrocytes, leucocytes, urothelial cells, and epithelial cells), bacteria, fungi and non-cellular components including urates and mucus filaments [36]. Thus, it is a non-inert fluid and residual cellular or enzymatic activities could significantly change the metabolic composition of the samples. It is, therefore, necessary to remove cells and bacteria and/or to quench the ongoing enzymatic or metabolic activities in urine samples.

#### 2.3.1. Timed vs. 24-Hour Collection

The first main consideration in urine collection is to choose the appropriate sampling time: 24-h collection or timed collection. It has been shown that there is a large variability depending of the collecting time (day vs. night, morning vs. afternoon) caused by the circadian rhythm regulating the energy metabolism and the gut microbiota metabolism and also due to a difference in physical activity and feeding state [37]. Therefore, 24-h sampling will be preferred if one aims at eliminating the day-time variability in metabolic profiles. Another advantage of 24-h sampling might be that it minimizes variation in urine concentrations compared to timed samples. Indeed, unlike blood where metabolite concentration is tightly maintained, urine concentration can vary drastically from sample to sample, thereby influencing the urine metabolome. In a recent review, Stevens et al. propose that pre-analytical normalization of urine (for e.g., to osmolarity) may improve the reliability of metabolomics analyses [38]. However, 24-h sampling is not always feasible, especially in humans. In rodents, specific individual metabolic cages or use of hydrophobic sand are required, in which mice are isolated and therefore mildly stressed [39,40]. 24-h sampling might also not be appropriate. For example, a timed sampling is needed to study a time-related trend and a kinetic sampling can be done to monitor the evolution of a targeted compound or the overall effect on the metabolism after drug or nutrient intake. In timed sampling, the time of collection is a very important point to ensure the reproducibility and quality of the study.

#### 2.3.2. Sample Collection

The most commonly used preservation methods are filtration, centrifugation or addition of bacteriostatics. Saude et al. showed that spinning urine samples at 112 g for 10 min was less effective in conserving the metabolome composition than filtration through a 0.22 μm filter [41]. Bernini et al. have shown that a mild pre-centrifugation (between 1000 and 3000 g) combined with filtration is the safest way to avoid contamination of the metabolic profiles attributed to bacterial removal without leading to an additional contamination due to cell damages or breaking (higher centrifugation speed induced partial breaking of cells and lower centrifugation was not effective to eliminate bacteria) [36]. Boric acid and sodium azide (NaN_3_) are the two most commonly used antimicrobial preservatives. It has been shown that the addition of 200 mM of boric acid or 10 mM of NaN_3_ for 24-h samples or 2–20 mM of boric acid or 0.1–1 mM of NaN_3_ for a timed sample are equally efficient to prevent bacterial overgrowth [42]. Nevertheless, boric acid is rarely used, as it induces formation of chemical complexes with endogenous metabolites [43]. Bernini et al. compared the use of NaN_3_ to a 0.2 μm filtration, and showed the latter to be superior for sample stability over time due to bacterial removal [36].

To summarize, filtration showed superior ability to preserve the urinary metabolites during storage in comparison with unfiltered samples. Moreover, the metabolic profiles of centrifuged samples are more stable than non-centrifuged samples after one week storage at −80 °C, with this effect being less severe in samples that are rapidly frozen in liquid nitrogen to avoid cell breaking. A mild pre-centrifugation plus a filtration seems to be the best method to avoid sample degradation.

#### 2.3.3. Sample Storage

For short-term storage, Gika et al. have shown that the storage of urine samples at 4 °C for up to 48 h maintained the metabolic integrity of the samples [44]. However, it is important to minimize sample storage at 4 °C as it has been shown that samples stored for more than 9 months will present an altered metabolome when compared to samples stored at −20 °C [45]. For long-term storage, metabolic profiles of urine samples stored at either −20 or −80 °C for 6 months did not show any significant differences [44]. This study, however, did not confirm whether or not the stored samples were identical to the original samples.

Freeze–thaw cycles have been shown to significantly modify the urine sample composition. Urine samples stored at −80 °C and thawed twice a week for 4 weeks (8 freeze–thaw cycles) indeed displayed a reduced metabolic stability in comparison to non-thawed ones stored at the same temperature. Metabolites deriving from bacterial metabolism (acetate, benzoate, succinate) increased [41]. Trivedi et al. showed that urinary metabolic profiles could be maintained only up to 3 freeze–thaw cycles using HILIC (Hydrophilic interaction liquid chromatography) mass-spectrometry [45].

### 2.4. Blood

Collecting blood is slightly more invasive than collecting urine, and the metabolic profiles of blood fractions provide a different, but complementary, metabolic information compared to the ones obtained with urine. Blood metabolic profiles are dynamic and vary continuously in response to changes in gene expression or changes induced by exogenous metabolites such as those provided by nutrients or drugs. Blood metabolic profiling is therefore widely used to study the dynamic variations of the endogenous metabolism in response to drug or food intake. Disruption in plasma metabolic profiles arises when homeostatic function is impaired. Serum and plasma are the most commonly used matrices, but other matrices do exist, such as platelet-free plasma (PFP), platelet-rich plasma (PRP) and whole blood, this latter receiving a growing interest.

Blood consists of two main components: plasma, which is a clear extracellular fluid containing clotting factors, proteins, glucose, minerals, and gases; and cellular elements, which are made up of blood cells (white blood cells, red blood cells) and platelets. Serum is the liquid fraction of whole blood, obtained by allowing the sample to clot naturally followed by a centrifugation step. The resulting supernatant is serum free of cells and of clotting factors such as the fibrinogen proteins. Plasma is prepared by collecting the whole blood into anticoagulant-treated tubes followed by a centrifugation step at 4 °C to separate blood cells. The supernatant designated as plasma is then immediately transferred into a clean tube. Plasma is a mixture of platelets, proteins, nutrients, hormones and gases. In some studies, further identification was given by naming it platelet-poor plasma (PPP) in opposition to platelet-free plasma or platelet-rich plasma by adding one or more additional centrifugation steps. Depending on the aim of the experiment, for example, if one wants to take into consideration the influence of growth factors or cytokines released by the platelets, the platelet content of the sample has to be carefully accounted for. Various manual, semi-automatic, and fully automated commercial systems have become available to prepare PFP, PPP and PRP [46].

#### 2.4.1. Sample Collection

Several studies have addressed a direct comparison of plasma vs. serum and have been recently reviewed [38]. The conclusions of this review highlight that both matrices are appropriate for blood metabolomics with minor differences between them.

The metabolomics analysis of serum is known to present a higher sensitivity of metabolites compared to plasma due to the lack of big particles. However, its processing time has the disadvantage of introducing variations due to enzymatic conversion and degradation processes, and to influence the metabolite composition [47]. Moreover, the reproducibility of serum is not as good as that of the whole blood because hemolysis can occur during collection or processing, leading to the presence of free hemoglobulin in the samples that influences the metabolic profiles [48].

In comparison, there is a better reproducibility in plasma due to the absence of the blood-clotting step. Moreover, it has been suggested that the absence of platelets and the lower protein content could be beneficial to small molecule analysis, because of a reduced competition [49]. For plasma preparation, the choice of anti-coagulant addition is critical and needs to be carefully accounted for before sample collection. Several anticoagulant collection tubes are available. The three most common additives are: heparin, ethylene diamine tetra acetic acid (EDTA) and citrate. They have often been compared with opposing conclusions depending on the analytical platform used. Actually, additives found in collection tubes can affect the ionization process during the MS run, thereby suppressing metabolite ionization and/or introducing interfering peaks. Bari et al. have compared heparin, EDTA and citrate anticoagulants using an untargeted UPLC-MS analysis. They noticed subtle metabolite differences between the different plasma preparations mainly due to ion suppression or enhancement caused by citrate and EDTA. Heparin did not cause interferences and was therefore recommended by the authors [50]. On the contrary, Yin et al. analyzed heparin, citrate, and EDTA collection tubes using a non-targeted LC-MS approach and they noticed that heparin led to chemical noise in the mass spectra. Citrate and heparin showed few additional signals. They recommended avoiding heparin, preferring EDTA [48]. As for NMR analysis, heparin is usually recommended, as EDTA, citrate and other stabilizers give additional signals in the NMR spectra [51]. The choice of collection tube for plasma preparation is therefore critical, should be consistent throughout the experiment and should be adapted according to the analytical platform used for subsequent analysis.

#### 2.4.2. Sample Preparation

After collection, samples should be quickly stored on ice. The time between collection and cell separation should be long enough to allow complete clot formation but short enough to avoid compositional changes. In general, it is recommended that the time before separation of blood cells should not exceed 30 min to minimize further metabolism or active and passive transport of analytes between the intra- and extracellular compartments. As for urine, whenever possible, samples should be stored as aliquots, allowing the use of fresh samples for each experiment and avoiding the introduction of bias due to repeated freeze–thaw cycles.

#### 2.4.3. Sample Storage

It is well established that serum and plasma contain high levels of enzymes, that are efficiently active at 37 °C. A reduced temperature decreases enzymatic activity, but it should be noted that this activity is not completely inhibited until temperatures below −56 °C are reached [52]. Lipids and lipoproteins are especially sensitive due to lipase activity [35]. Small changes have been observed in the plasma metabolic profiles after one-month storage at −20 °C [35,53], while storage at −80 °C for 4 years had minimal effects [54,55].

Data regarding the number of freeze–thaw cycles acceptable are variable [42,44,46]. Unfractionated serum samples can be stored frozen for later quantitative lipid analysis as minor effects occur on quantitative lipid composition for most of the biologically relevant lipid species in humans, even with one to three freeze–thaw cycles. At the opposite freezing prior to lipoprotein fractionation significantly introduce a large variability in high-density lipoprotein and low-density lipoprotein cholesterol as well as in very low-density lipoprotein free fatty acids compared with fresh samples: density-based fractionation should preferably be undertaken in fresh serum [39].

### 2.5. Feces

Feces represents a growing interest in metabolomics studies, as fecal metabolic profiles reflect the metabolic interplay between the host and its gut microbiota [56]. The fecal metabolome has been shown to largely reflect gut microbiota composition in humans (explaining on average 67.7% of its variance), and is considered to be a functional readout of the microbiome [26]. Despite the rising popularity of fecal metabolomics, the methods for collecting, preparing and analyzing fecal samples are still far from being standardized. In a recent review, Karu et al. provided the state of knowledge with regards to the protocols and technologies in human fecal metabolite analysis [57]. They also present a comprehensive database that contains over 6000 identified human fecal metabolites, thereby highlighting the potential richness of the information contained in the metabolomics analysis of fecal samples. While the first metabolomics study of human feces used headspace GC-MS to study volatile organic compounds (VOCs) [58,59], it is now recognized that the majority of fecal metabolites are non-volatile [57].

The largest part of stool is made up of water (60–80%, depending on fiber intake), while the dry matter contains bacteria (both alive and dead, representing 25–54% of biomass) derived from the gastro-intestinal microbiota, colonic epithelial cells, macromolecules, undigested food residues, and thousands of metabolites including sugars, organic acids and amino acids, that constitute the fecal metabolome [60]. The latter includes both compounds derived from the metabolic activity of the gut microbiota and various host endogenous metabolites such as signaling peptides or bile acids [61].

#### 2.5.1. Sample Selection

Timed vs. multiple-timed sampling: Much information contained within the fecal metabolome derives from dietary inputs and biochemical events that have occurred during their digestion. Thus, there is inherent variability in fecal samples depending upon feeding state and bowel activity. Both the gut microbiota composition and metabolic activity have been shown to be highly circadian [62,63]. Therefore, as for urine, it can be expected that timed collection vs. 24-h collection will provide different information. In animal studies, both timed [64] and 24-h [65] fecal sampling are commonly used for specific biochemical assays such as sterol and bile acid profiling; however, to our knowledge, no direct and systemic comparison of timed vs. 24-h fecal metabolome has been performed yet. In humans, it might not be feasible or relevant to collect 24-h samples. However, it was shown that the ^1^H-NMR-based fecal metabolic profiles from single time samples greatly varied within one individual (day to day variation), and multiple day sampling and pooling has been proposed to minimize errors arising from day to day variation [66].

Presence of blood in stools: Gut bleeding is a clinically prevalent phenomenon associated with many gastro-intestinal diseases. The impact of blood in stool on the fecal metabolome has been shown to be minimal if the level of contamination is low (occult blood). However, gross (visible) blood in the fecal sample significantly contaminates the fecal metabolome [67]. Therefore, Karu et al. recommend visually inspecting samples and considering excluding the fecal samples or portions of fecal samples with gross blood [57].

#### 2.5.2. Sample Collection

Feces collection presents the advantage of being non-invasive. In animals, fecal samples can be directly obtained from the intestine after euthanasia or collected from alive subject and pooled if necessary. Twenty-four hour feces can easily be obtained using metabolic cages; however, a mild but significant increase in fecal output was observed when housing rats in metabolic cages [39]. In humans, fecal samples can be directly collected in a falcon tube, in a plastic container, in a sterile bag [68,69], or in special anaerobic pouch systems [70]. Stabilizing solutions such as nucleotide stabilizers present in some stool collection kits should be avoided because they interfere with subsequent metabolomic analysis. Similarly, if stool samples are to be collected prior to colonoscopy, Bezabeh et al. recommend collection before patients start taking the solutions used for colonoscopy [71]. Indeed, most of these solutions contain polyethylene glycol, which produces strong interfering signals in the ¹H-NMR spectrum.

Sample type and amount have to be decided beforehand and standardized. Samples for metabolomics study can be intact (crude) feces (usually for analysis of VOC), fecal water (the water fraction of an intact feces, obtained by ultracentrifugation of the stool), or a fecal aqueous extract (obtained after the addition of an aqueous buffer or of water to the stool, followed by homogenization and centrifugation). Fecal water generates a different metabolic coverage from feces and GC-MS analysis from crude feces samples yielded detection of more peaks than analysis of fecal water samples [67]. Stool samples are highly heterogeneous, and the topological position from the fecal sample has been shown to influence the fecal metabolome [72]. Therefore, it is sometimes recommended to collect as much sample as possible and to homogenize it before preparing aliquots, enabling a non-selective and more reproducible method [57,71]. The metabolic stability of aqueous extracts was shown to be higher than that of crude feces samples and it was recommended that fresh samples should be refrigerated and aqueous extraction conducted ideally within 1 h (and not longer than 24 h) after collection before aliquoting and freezing [57,72].

Exposure to aerobic conditions and room temperature might quickly change the fecal metabolome due to microbial fermentation. Some researchers therefore place their fecal samples in an anaerobic chamber within 10 min of collection [73]. Couch et al. investigated the differences between home-based self-collection (ex vivo) samples and lab-based endoscopic collection (in vivo) samples in healthy subjects [74]. Using GC-MS, they found modest differences in the overall chemical distribution with a slight bias toward oxidized metabolites in the ex vivo samples. Further investigation revealed significant differences in the VOC metabolomes between the two groups. The effect of post-collection storage is much more drastic in fecal samples than in any other biological matrix, and most researchers store their fecal samples at 4 °C or lower immediately after collection. Fecal metabolites have indeed been shown to be highly unstable upon several storage conditions. For example, using GC-MS analysis, Phua et al. showed that, over 268 analytes, only 28% remained stable when crude feces were stored for one day at 4 °C, and this declined to 10% at room temperature (29 °C) [67]. Immediate cooling of fecal samples is not always feasible, especially when human feces are collected at home or in clinics. Using LC-MS, Loftfield et al. compared several methods allowing preserving sample quality and demonstrated that crude fecal samples collected in 95% ethanol were stable for up to 96 h at room temperature [75]. Interestingly, these ethanol-preserved samples exhibited a metabolic profile more akin to fresh samples compared to immediately frozen-feces. This protocol represents an interesting alternative when immediate freezing of samples is not possible.

Water content in feces is variable (60–85% in human) and this can sometimes create a bias to compare experimental groups. Immediately after collection, or before metabolites extraction, it is possible to lyophilize or freeze dry the samples to remove the water present in the feces. This improves sample weight precision, reduces bias due to the volume of solvent and/or derivation reagents added to samples during the metabolite extraction steps and allows quantitative metabolite data to be given in units per dry matter weight. This latter point is especially important for meta-analysis of metabolomics results and to establish reference levels for clinical use. While working with dried samples is less laborious, more reproducible and prevents bacterial growth, it also results in a loss of detected metabolites, especially VOCs. Indeed, the effect of lyophilization has been compared with the use of fresh sample and results in a decrease in short chain fatty acids [76,77]. Therefore, Karu et al. suggest that, unless volatile compounds are specifically targeted for quantification, fecal samples should be dried and weighed prior to storage or analysis. However, some researchers recommend not using it to minimize the number of preparation steps and guarantee quantifiable levels of short-chain fatty acids among others.

#### 2.5.3. Sample Storage

While the analysis of fresh fecal samples is therefore recommended, the use of frozen samples could be more convenient. Two NMR studies investigated the effects of freezing on crude feces and/or aqueous fecal extracts and showed higher levels of several amino acids (namely branched-chain and aromatic amino acids) and glucose upon a single freeze–thaw cycle [72,76]. This observation has been confirmed using GC-MS analysis. Phua et al. indeed observed that only 33% of the initial metabolites remained after one cycle, and 18% after two and three cycles [67]. In the same study, the metabolites also exhibited a poor stability at −80 °C, with only 24% of stable metabolites after 1 and 6 weeks of storage. The identity of the impacted metabolites was not given.

To summarize, although the immediate processing of fresh fecal samples is recommended, the use of frozen samples is often much more convenient. According to the last recommendations [57] and our present review, it seems that good practices would require fecal samples to be homogenized and aliquoted prior to freezing, while minimizing handling time at the lowest temperature possible. Very importantly, freeze–thaw cycle and storage duration should be minimized.

### 2.6. Tissue

Most metabolomics studies are based on non-invasive or minimally invasive sample types, such as blood, urine and feces. However, tissue analyses are also important, as the tissue represents the first place where the metabolic changes owing to a disease take place. Metabolomic studies have been conducted on almost every tissue (liver, intestine, muscle, adipose tissues from various locations, whole brain or selected brain areas, etc.). Most tissues are not homogenous. For example the liver has five different lobes, and within them the portal and periportal vascular regions are known to display different levels of some enzymatic systems such as those involved in glycolysis and gluconeogenesis [78,79,80]. Even when the tissue is composed of the same cell types, regional differences in composition may still exist. Such factors may result in increased biological variability, which should be taken into account during sampling [81].

#### 2.6.1. Sample Collection

There are several critical points to be aware of during the handling step of tissues to avoid bias due to the collection procedure. The first important consideration is to ensure sample collection homogeneity throughout the experiment by always collecting samples from the same region to avoid bias due to biological variability. Whenever possible, it is also advisable that the same person collects the tissue samples throughout the experiment to minimize variability. Furthermore, to avoid contamination with blood metabolites, it is possible to wash the samples with cold deionized water or PBS after collection [82]. In some cases, saline in D_2_O or PBS is injected into the organ-associated artery before the collection to remove residual blood in organs [83]. One should also be aware that contaminant signals can result from the anesthetic used during the experiment, or even from surgical instrument cleaning solutions, especially ethanol that produces additional signals in the ^1^H-NMR spectra, thereby masking signals from endogenous metabolites.

#### 2.6.2. Sample Storage

To obtain useful global metabolic profiles, sampling must be performed as rapidly as possible and samples should be either processed immediately or snap-frozen in liquid nitrogen to minimize further metabolism [84]. A good practice is to cut each sample into small pieces and to freeze the organs as rapidly as possible. For large prospective biobanking studies, this might not always be feasible and tissue samples might undergo several cycles of “storage-near-retrieval” due to storage constraints of adjacent samples for example. Testing different scenarios of storage–retrieval cycles in human liver tissues demonstrated that storage temperature affected metabolite concentrations only little, while there was a linear dependence on the number of temperature change cycles [85]. Metabolic changes induced by thawing were shown to be almost identical for all organs, with a marked increase in overall metabolite levels caused by increased protein and cell degradation [86].

### 2.7. Cells

Metabolomics analysis of cultured cells has emerged as an important technology for studying cellular biochemistry that provides an instantaneous snapshot of ongoing cellular metabolism. The major bottlenecks associated with metabolomics cell samples preparation workflow are efficient sampling, quenching and metabolites extraction in order to preserve the internal metabolite signatures [87].

#### 2.7.1. Sample Preparation

Metabolomics analyses have been performed on a broad range of adherent cell numbers, ranging from 1 × 10^4^ up to 4 × 10^7^ cells [88]. Depending of the cells and the technology used to process the cell extractions, the seeding number must be optimized in order to get enough signal in NMR and/or MS, to be able to detect small metabolites that are present at low concentration but that still may be important for biological purposes, especially when global metabolomics is performed. Regarding the hepatic HepG2 cells for instance, tests have been performed to optimize the number of cells needed to obtain NMR metabolic fingerprints allowing to detect and identify the metabolite content in the cells. The number of 10^6^ cells was selected, as this seeding allowed the detection of the subtle modulations occurring after exposing the cells to xenobiotics such as estradiol or bisphenol A [18]. For cell extracts, it is usually recommended to work in very low volumes and concentrate the extract in a maximum of 50 uL. Microvolumes are easily handled using MS-based metabolomics or can be transferred to NMR microtubes or capillary tubes for analysis.

Culture media composition is also important to consider as some media contain interfering anions such as Cl^−^, SO_4_^−^, and PO_4_^−^, and depending on cultivated cells, amino acids, Good’s buffers, organic acids, and complex biological mixtures such as fetal bovine serum. These components can cause substantial electrospray ionization suppression, additional signals in ¹H-NMR spectra or contaminate the intracellular metabolite pool [88]. With regard to NMR-based metabolomics, culture media that contain HEPES should be avoided or carefully removed, since HEPES gives many broad additional signals. For these reasons, cells have to be separated from the medium before analysis. The washing step is therefore very important and should be performed with caution to effectively remove all the extracellular media components. Kapoore et al. (2017) performed five different washing protocols on the breast cancer cell line MDA-MB-231, using either PBS or distilled water. Based on a few metabolites detected by GC-MS, these authors suggested that in their conditions, a single washing step with PBS followed by quenching using 60% methanol supplemented with 70 mM HEPES (−50 °C) was the best condition for minimizing intracellular metabolites leakage. More recently, three-dimensional multicellular tumor spheroids have been used to perform metabolomics analysis. The washing steps consisted of a rapid washing of the spheroids directly on the cultivation plate only once using PBS [89].

Another crucial point for reducing the variability and improving the quality of sample preparation is to quench the cells before the extraction. This procedure aims at stopping cellular metabolism to prevent the turnover of metabolites, maintaining the metabolite concentrations at their physiological levels. Thus, the quenching method should immediately stop all cellular enzymatic activities or cellular changes in metabolite concentrations, without changing the cell environment, since metabolites are very sensitive to any variation of their environment. As for the other points mentioned above, several protocols have been tested and used by scientists and were improved over time. Previously, metabolic quenching and extraction were performed using drastic culture conditions changes (acidic condition, high temperature) to inactivate all enzymatic activities, but taking into account the loss of heat-sensitive and pH-sensitive compounds [90]. Some papers reported quenching protocol using liquid nitrogen, for metabolomics studies, after detaching cells by trypsination [91]. In this case, cells are quenched and extracted at the same time [92]. This method substantially modifies the intra-cellular metabolic profiles because trypsin can induce cell stress, structural and protein disruption during cell detachment and consequently metabolite leakage [93]. Another method has been proposed to isolate the cells using filtration [88]. However, both filtration and centrifugation expose cells to a mechanical stress that can also modify the metabolic profiles. Recently, another method has been proposed to better preserve cell metabolism: after carefully removing the medium, cells are washed with PBS or deionized water at room temperature or at 37 °C. Finally, the cellular metabolism is quenched by adding liquid nitrogen or ice-cold methanol into the dishes. Cells can be stored at −80 °C or scraped with a cell lifter to be directly extracted. The solvents used for the quenching and metabolite extraction step is one critical step to anticipate, depending of which type of metabolites are targeted. It is admitted that up to now the choice of solvent was adapted to specific metabolite extractions. Usually, a mix of different proportions of solvents is used to quench, extract the cells and collect the metabolites. Mainly, methanol or acetonitrile are used as organic solvents, supplemented by water or acidified water [94]. Different ratios of organic solvents and water have been documented, such as methanol:water 50:50 (*v*/*v*) to perform global metabolomics [93] or acetonitrile:water 70:30 (*v*/*v*) to perform targeted amine profiling [95]. Some protocols include only a cold mix freshly prepared of acetonile and deionized water to seek polar metabolites [96]. The choice of methanol, acetonitrile and water, even at different ratios, is efficient to recover a large panel of metabolite families such as amino acids, organic acids, nucleotides precursors, sugars ad sugar alcohols [97]. Potassium hydroxide or perchloric acid have also been used, but the results were less reproducible and less efficient for extracting nucleotides, sugars, sugar phosphates and organic acids. These authors concluded that acidic and alkaline extractions did not suit the requirements for a global metabolome analysis [97]. Research is ongoing to optimize conditions in order to get as many metabolites as possible from the same sample extracts using appropriate mix of solvents.

#### 2.7.2. Sample Storage

After quenching and metabolite extraction, cell extracts are usually snap frozen in liquid nitrogen and quickly stored at −80 °C until further analysis to prevent metabolite degradation. Similarly to animal tissues, freeze–thaw cycles must be avoided and the thawing step should be performed on ice to increase gradually the temperature of the samples.

## 3. Conclusions

Metabolomics offers detailed insights into the metabolic phenotype of the liver and each of the associated organs and biofluids (including the gut microbiota). It is, therefore, nowadays one of the most promising tools in systems biology in hepatology, and is expected to help especially in non-invasive biomarker discovery and identification of biological pathways operating in the liver in physiology and pathology. Metabolomics studies have therefore been performed in a variety of biological matrices to study liver functions. Depending on the research question, several points have to be carefully considered before analytical handling of the samples, including: type and time of sampling, sampling conditions, quenching of ongoing metabolism, use of preservatives, aliquoting and storage conditions. SOPs vary according to the biological matrices used but aim to enhance metabolite recovery and stability to optimize metabolic pathways investigations in liver functions. This raises the question of international consensus protocols and international committees working on continuous improvement of standardized pre-analytical issues.
